# Profiling of suppressive immune subsets in metastasis negative and positive sentinel lymph nodes from patients with HER2- breast cancer

**DOI:** 10.1186/2051-1426-2-S3-P237

**Published:** 2014-11-06

**Authors:** Rieneke van de Ven, Kim van Pul, Shaghayegh Aliabadi, M Petrousjka van den Tol, Daniel Haley, Raina Tamakawa, Ronald J Vuylsteke, Hein B Stockmann, Julie L Cramer, Walter J Urba, Bernard A Fox, Tanja D de Gruijl

**Affiliations:** 1 VU University Medical Center / Department of Medical Oncology, Amsterdam, Netherlands; 2 VU University Medical Center / Department of Surgical Oncology, Amsterdam, Netherlands; 3 Robert W. Franz Cancer Research Center at the Earle A. Chiles Research Institute, Providence Cancer Center, Portland, OR, USA; 4 Kennemer Gasthuis / Department of Surgery, Haarlem, Netherlands

## Background

Since it is the site of initial immune activation and priming of antigen-specific T cells, as well as the first location to which tumor cells metastasize, our research focuses on understanding the immune status and tumor-induced suppression within breast cancer (BrCa)-draining sentinel lymph nodes (SLN).

## Methods

Suppressive immune subsets were profiled by multi-color flow cytometry in two different BrCa SLN cohorts. In the first cohort, collected at the Providence Cancer Center (08/11-07/13), we looked at frequencies of HLA-DR- CD14+ myeloid cells and their expression of co-stimulatory and inhibitory receptors. Four metastasis+ SLN and 11 metastasis- SLN were assessed in this cohort. SLN in the second cohort were collected at the VUmc in Amsterdam and Kennemer Gasthuis in Haarlem (10/13-06/14) and contained 2 metastasis+ and 7 metastasis- SLN. In this cohort the frequencies of HLA-DR- CD14+ cells as well as CD25_hi FoxP3+ T regulatory cells (Treg) were analyzed. Since all tumors corresponding to the metastasis+ SLN turned out to be HER2 negative, only metastasis- SLN from HER2- tumors were included. Apart from 2 tumors in the Providence cohort, all tumors did express progesterone and/or estrogen receptors.

## Results

Elevated frequencies of HLA-DR- CD14+ immature myeloid cells could be detected in the metastasis+ SLN in both cohorts. This difference was statistically significant for the Providence cohort (p < 0.0001) (3.8 fold increase). No differences were observed for expression levels of the co-stimulatory molecules CD80 and CD86 or the inhibitory molecules PD-L1 and B7H4 on HLA-DR- CD14+ cells between positive or negative SLN and expression was low for all these markers. Due to the small number of metastasis+ SLN in the Dutch cohort statistics could not yet be performed, but a 2.3 fold increase in HLA-DR- CD14+ cells was seen. In this cohort, frequencies of Treg were found to be 4-fold higher in the metastasis+ SLN compared to metastasis- SLN (0.31 ± 0.22 vs. 1.22 ± 0.24 Treg of CD4 T cells). Moreover, a significant correlation was observed between the frequencies of HLA-DR- CD14+ immature myeloid cells and the frequencies of Treg in these BrCa SLN (r = 0.718, p < 0.01).

## Conclusion

Our data suggest that tumor-derived factors negatively influence both the myeloid and the lymphoid compartments within SLN draining HER2 negative breast cancers.

## Consent

Written informed consent was obtained from the patient for publication of this abstract and any accompanying images. A copy of the written consent is available for review by the Editor of this journal.

